# Crosstalk Between the Gut Microbiome and Bioactive Lipids: Therapeutic Targets in Cognitive Frailty

**DOI:** 10.3389/fnut.2020.00017

**Published:** 2020-03-11

**Authors:** Liliana C. Baptista, Yi Sun, Christy S. Carter, Thomas W. Buford

**Affiliations:** ^1^Division of Gerontology, Geriatrics and Palliative Care, Department of Medicine, University of Alabama at Birmingham, Birmingham, AL, United States; ^2^Integrative Center for Aging Research, University of Alabama at Birmingham, Birmingham, AL, United States

**Keywords:** bioactive lipids, gut microbiome, cognitive frailty, eicosanoids, phospholipids, sphingolipids, specialized pro-resolving mediators, endocannabinoids

## Abstract

Cognitive frailty is a geriatric condition defined by the coexistence of cognitive impairment and physical frailty. This “composite” aging phenotype is associated with a higher risk of several adverse health-related outcomes, including dementia. In the last decade, cognitive frailty has gained increased attention from the scientific community that has focused on understanding the clinical impact and the physiological and pathological mechanisms of development and on identifying preventive and/or rehabilitative therapeutic interventions. The emergence of gut microbiome in neural signaling increased the interest in targeting the gut–brain axis as a modulation strategy. Multiple studies on gastroenteric, metabolic, and neurodegenerative diseases support the existence of a wide bidirectional communication network of signaling mediators, e.g., bioactive lipids, that can modulate inflammation, gut permeability, microbiota composition, and the gut–brain axis. This crosstalk between the gut–brain axis, microbiome, and bioactive lipids may emerge as the basis of a promising therapeutic strategy to counteract cognitive frailty. In this review, we summarize the evidence in the literature regarding the link between the gut microbiome, brain, and several families of bioactive lipids. In addition, we also explore the applicability of several bioactive lipid members as a potential routes for therapeutic interventions to combat cognitive frailty.

## Introduction

Cognitive frailty is an intermediate neuro-degenerative state defined by the coexistence of physical frailty syndrome and cognitive impairment in the absence of a clinical diagnosis of Alzheimer's disease or other types of dementia (1). This composite phenotype is associated with an increased risk of adverse health outcomes such as falls, disability, hospitalization, institutionalization, and mortality (2, 3). Older adults with cognitive frailty also have a higher risk of dementia, limitations in the activities of daily life, and lower quality of life (3).

Epidemiological evidence estimates that the prevalence of cognitive frailty ranges from 1 to 22% (11–22% in clinical settings and 1–4% in community settings), varying according to the conceptual definition used to categorize it (e.g., the Fried phenotype or the deficit accumulation model) (4). As the “Baby Boomer” generation passes the age of 65, the incidence of people living with cognitive frailty is expected to increase in the coming years (4–6). This projected growth is expected to have a significant impact on multiple social structures, including on the individual, caregivers, families, and clinical settings, as well as on healthcare systems. Thus, it is crucial to identify therapeutic strategies that can prevent, rehabilitate, or even reverse cognitive frailty. Notably, cognitive frailty as an intermediate disease stage may be an important target for primary and secondary prevention of several neurodegenerative diseases (4, 7).

From a pathophysiological point of view, multiple factors are involved in the onset of cognitive frailty, including low-grade chronic inflammation, hormonal dysregulation (i.e., hypogonadism and hypovitaminosis D), malnutrition (e.g., low diet quantity/quality), and anorexia of aging (3, 4). Pathological changes in the vascular, neuro-musculoskeletal, and metabolic systems also increase the risk of cognitive frailty, cardiovascular disease, cardiac dysfunction, depression, sarcopenia, insulin resistance, and dyslipidemia (3, 4). These biophysiological factors and disorders are associated with the onset of cognitive frailty but also with the imbalance of the gut microbiome, the microorganism complex that inhabits the gastrointestinal tract (8, 9).

**Figure 1:**
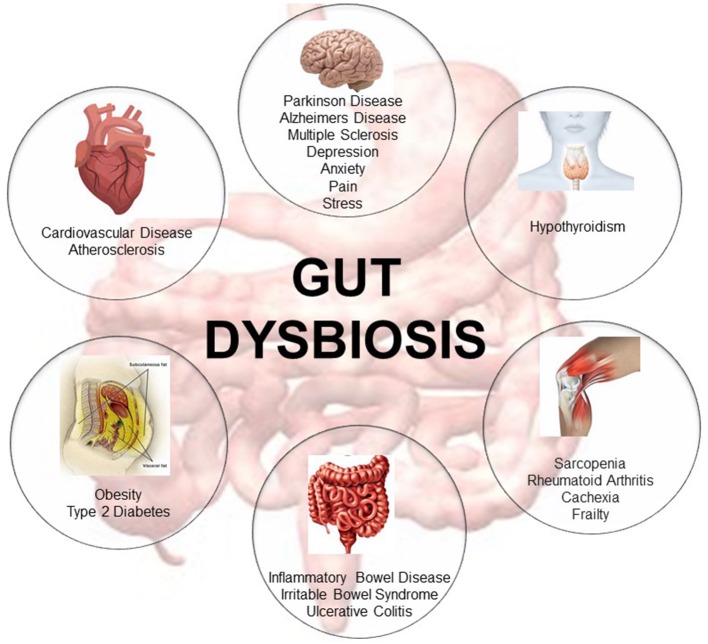
Representative description of the metabolic diseases, gastrointestinal disorders, neuromusculoskeletal conditions, endocrine pathologies, neurodegenerative, and cardiovascular diseases associated with gut dysbiosis [adapted from Buford (10)].

In the last decade, extensive research has focused on determining the role of the gut microbiome in host health (10). Many studies associate gut dysbiosis—the loss of microbe species richness and increased interindividual variability (9)—with the onset of several pathological diseases involving not only the gastroenteric system but also distant organs (3, 11–13). Changes in gut microbiota composition are associated with aging (14), with indices of age-related inflammation (14, 15), and with a higher risk of inflammatory bowel disease and irritable bowel syndrome (16). In addition, other extra-gastrointestinal diseases such as atherosclerosis, metabolic syndrome, hypertension, type 2 diabetes, and neuromusculoskeletal diseases (i.e., cachexia and frailty) are also associated with gut dysbiosis (3, 11, 17–21). Imbalance of the gut microbiome is also associated with neurodegenerative diseases such as Parkinson's and Alzheimer's disease (22–25) and multiple sclerosis (26, 27) (Figure 1). Remarkably, some of these diseases are involved in the onset of cognitive frailty (3, 4).

**Figure 2:**
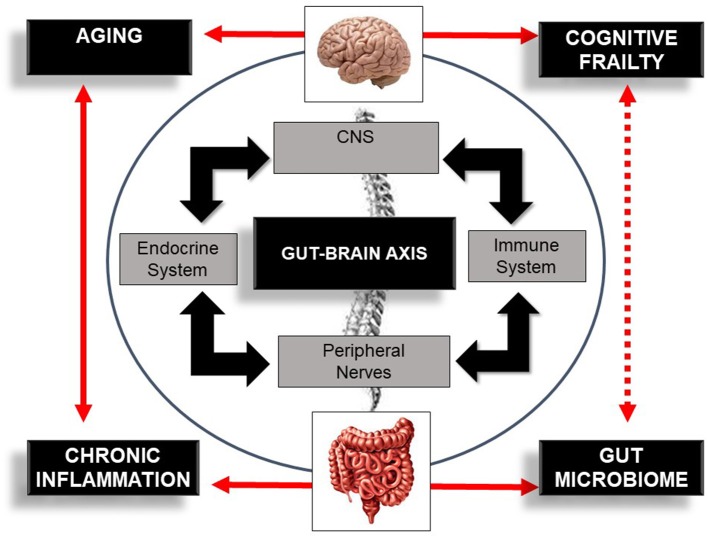
Schematic illustration of the bi-directional communication system—the gut–brain axis—comprising the central nervous system (CNS), immune system, endocrine system, and peripheral nerves. The figure also illustrates the bi-directional influence of aging, inflammation, and the gut microbiome in cognitive frailty. Bold lines indicate an established association between concepts. Dotted lines indicate a potential association between this bi-directional network, the gut microbiome, and cognitive frailty.

The link mediating gut dysbiosis and these multiple diseases is an integrative bidirectional communication system—the gut–brain axis (19, 28, 29). The gut–brain axis, which includes the central nervous system (brain and spinal cord), the autonomic and the enteric nervous system, and peripheral nerves (16), establishes an interdependency relationship between host-microbe and environment (30), influencing host health and longevity (31). This axis influences brain function but also the functionality and structure of a plethora of other distant organs (19, 28, 29) modulating host immune response (30) and host-cell proliferation and vascularization (32, 33), regulating intestinal endocrine functions and neurologic signaling (34). This system also modulates energy biogenesis (35) and the biosynthesis of vitamins and neuro-transmitters (36), metabolizes bile salts (37), reacts or modifies specific drugs, and eliminates exogenous toxins (38). Overall, the gut–brain axis is an important integrative system that modulates host immune function, metabolic balance, and resistance and resilience to infection (3, 11–13) and hence, is a potential target for managing cognitive frailty (Figure 2).

Host health and longevity are also influenced by a plethora of other intricate processes, networks, and cellular signaling mechanisms, including intestinal barrier permeability, elevated production of microbe metabolites (e.g., short-chain fatty acids), and an increase in gut peptides (3, 16, 39, 40). Notably, lipids—a diverse group of organic biomolecules—are essential elements in gut permeability as the major constituents of cell membranes and hence, in the modulation of the gut microbiome (41). Lipids also regulate multiple cell functions through intercellular and intracellular signaling mediators that are present in the brain and the enteric system (16). These signaling mediators are then released into the bloodstream, migrating to distant organs through the gut–brain axis (16), which has led them to earn the name bioactive lipids (42). Multiple bioactive lipids act “on demand,” exerting either pro- or anti-inflammatory actions on the gut microbiome and on the immune and central nervous system (3, 17, 43), influencing immune regulation, inflammation, and homeostasis (16, 41–43). Targeting the crosstalk between the gut microbiome and bioactive lipids may be crucial to control metabolism and inflammation, to regulate the gut–brain axis (11), and potentially to modulate cognitive frailty. Hence, the aim of this review is to summarize the evidence in the literature regarding the crosstalk between the gut–brain axis, the microbiome, and bioactive lipids. In addition, this review also discusses their relationship in cognitive frailty as potential therapeutic targets to counteract this aging phenotype.

## Crosstalk Between the Gut–Brain Axis, Microbiome, and Bioactive Lipids

To date, the characteristics of the gut microbiome in patients with cognitive frailty are undescribed. Nonetheless, evidence from multiple studies in chronic diseases involved in the onset of cognitive frailty supports the crosstalk between the gut–brain axis, microbiome, and bioactive lipids (3, 16, 44, 45). For instance, Claesson et al. (15) showed that a loss of diversity in the fecal microbiota composition in older adults with declining health status correlated significantly with frailty, co-morbidity, geriatric depression, and inflammation (15). When compared to community-dwelling older adults, the fecal microbiota of the elderly in long-stay care was significantly less diverse and was associated with decreased mobility and reduced nutritional variability (15). Diet and supplementation influence the formation and function of bioactive lipids and hence, the availability of ω-6 and ω-3 polyunsaturated fatty acid (PUFA) precursors (41). Evidence supports an inverse relationship between nutritional quality and the prevalence of Alzheimer's disease. Lower ω-3 PUFA intake is associated with increased risk of Alzheimer's disease (46), whereas, high ω-3 PUFA intake is associated with benefits in cognition and mobility and reduction in frailty incidence in non-demented older adults (47–49).

Among the metabolic diseases, Everard et al. (50) showed that the administration of *Akkermansia muciniphila*—a mucin-degrading bacterium that resides in the mucus layer—improves gut barrier function and gut peptide secretion, reduces metabolic endotoxemia, and decreases metabolic inflammation in obese, type 2 diabetic mice (50). These changes were associated with the increase in the intestinal levels of acylglycerols, a precursor of 2-Arachidonoylglycerol (2-AG), a bioactive lipid of the endocannabinoid family (50). The deletion of intestinal epithelial myeloid differentiation primary response gene 88—a signaling pathway of Toll-like receptors (TLR)—protected against obesity, diabetes, inflammation, and gut barrier disruption (51). These effects were mediated by the increase in the intestinal levels of arachidonoylethanolamide (AEA) and 2-AG, which, in turn, restored the production of antimicrobial peptides and intestinal regulatory T cells (51).

Several studies on gastroenteric diseases also support the crosstalk between the gut–brain axis, microbiome, and bioactive lipids. In an experimental model, comparing saturated vs. PUFA diet, mice fed with fish oil (rich in ω-3 PUFA) had increased levels of *Lactobacillus*, a bacteriun known to be associated with reduced inflammation and mucosal lesion scores in inflammatory bowel disease (52). These rodents also had increased levels of *Akkermansia muciniphila*—a bacteria associated with improved gut permeability and glucose metabolism—and had reduced fat mass gain and white adipose tissue macrophage infiltration (52). The administration of *Lactobacillus plantarum*—a strain of the *Lactobacillus* species—was also associated with protective effects in inflammatory bowel disease through the mediation of T cells (52). This mediation occurred via an apoptotic inhibition mechanism blocking the action of cyclooxygenase 2, an enzyme that drives the synthesis of several inflammatory members of the eicosanoid family. This process reduced the expression of several pro-inflammatory cytokines, including tumor necrosis factor-alpha (TNF-α), interleukin (IL)-1β, IL-6, forkhead box P3, suppressors of cytokine signaling 3, and TLR4, the receptor for the microbial endotoxin lipopolysaccharide (LPS) (52).

Despite the cumulative evidence supporting the protective effect of several species of bacteria in the crosstalk between the gut–brain axis, microbiome, and bioactive lipids, there is evidence that some strains have detrimental effects on the central nervous system. For instance, *Cyanobacteria* is associated with intra-neuronal protein misfolding and neuroinflammation, characteristics that are elevated in the brains of Parkinson's and Alzheimer's disease patients (53). This bacterium produces β-*N*-methylamino-L-alanine, an excitotoxin that activates metabotropic glutamate receptor 5, inducing the depletion of glutathione (16, 53). As a result, neurons and glial cells are unable to control the balance of reactive oxygen and nitrogen reactive species, increasing the oxidative stress gradient in the brain and leading to neurodegeneration. β-*N*-methylamino-L-alanine is also associated with the aggregation of proteins into amyloid fibers, a pathological mechanism found in some neurodegenerative diseases such as Alzheimer's and Parkinson's (16, 53). Several studies suggest that these pathological processes can be counterbalanced by the administration of several members of the endocannabinoid family (54–58). Palmitoylethanolamide and oleoylethanoloamide are among those bioactive lipid members and may provide protective effects in neuroinflammation, oxidative stress, and neurodegeneration (54–58). Overall, this body of evidence supports the crosstalk between the gut–brain axis and the microbiome, as well as their modulation by several bioactive lipid mediators. Given that many of these pathologic conditions are involved in the onset of cognitive frailty, the potential of the gut–brain axis, microbiome, and bioactive lipids as therapeutic targets in cognitive frailty can be hypothesized.

## Bioactive Lipids, the Gut Microbiome, and Inflammation

**Figure 3:**
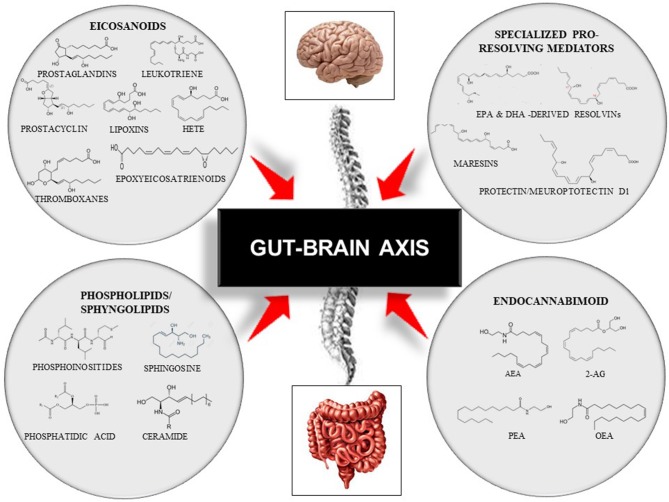
Representative scheme of the gut–brain axis and the modulation by four families of bioactive lipids: the classical eicosanoids, the phospholipids/sphingolipids, the specialized pro-resolving lipid mediators, and, the endocannabinoid system. This figure does not intend to be an exhaustive description of all lipid members or each family sub-member. 2-AG, 2-Arachidonoylglycerol; AEA, Arachidonoylethanolamide; DHA, Docosahexaenoic Acid; EPA, Eicosapentaenoid Acid; HETE, Hydroxyeicosatetraenoids; OEA, Oleoilethanolamide; PEA, Palmitoylethanolamine.

Lipids are organic biomolecules with a wide variety of compounds and functions. To date, its categorization is still a source of debate within the scientific community. Due to the lack of a consistent classification, the Lipid MAPS consortium developed a classification system based on lipid chemical and biochemical properties, dividing them into eight categories: fatty acyls, glycerolipids, glycerophospholipids, sphingolipids, sterol lipids, prenol lipids, saccharolipids, and polyketides (59). Each of these categories is then sub-divided into its own subclassification hierarchy. In this review, we only focused on four bioactive lipid families due to their roles in immunity and inflammation and their associations with several chronic diseases that are involved in the onset of cognitive frailty (3, 4). Thus, we focused on: (i) the eicosanoids, the inflammation “fire starters”(16); (ii) the phospholipids and sphingolipids, the major membrane constituents; (iii) the specialized pro-resolving lipid mediators, the acute immunoresolvents; (iv) the endocannabinoid system (16, 41, 60, 61) (Figure 3).

## Eicosanoids

The classical eicosanoids are derived from the ω-6 PUFA arachidonic acid (AA) that is then used as the substrate for different oxygen-incorporating enzymes that together drive the synthesis of other heterogeneous and pleiotropic molecules. For example, cyclooxygenases (COX) 1 and 2 drive the synthesis of prostaglandins (PG), prostacyclins, and thromboxanes. The 5-,12-, 15-lipoxygenases (LOX) synthetize leukotrienes, hydroxyeicosatetraenoids (HETE), and lipoxins. Lastly, the P450 epoxygenase generates HETE and epoxyeicosatrienoids, as fully reviewed elsewhere (41, 62).

Eicosanoids are involved in many physiological and homeostatic processes, including the control of vascular tone, platelet aggregation, and pain perception (41), although they are more renowned by their role in the immunity and inflammation process (41). They act as “initiators” of the inflammation process, amplifying or reducing the inflammation responses and coordinating leukocyte recruitment, cytokine and chemokine production, antibody formation, cell proliferation and migration, and antigen presentation (43, 63). Some eicosanoids like PGs, lipoxins, and leukotrienes also play an important role in the maintenance of mucosal integrity (64). When the gut barrier integrity is lost, these mediators drive major inflammatory processes (64), amplifying the expression of several pro-inflammatory cytokines (e.g., TNF-α, IL-1β, and Il-6) that have deleterious effects on the gut–brain axis (41, 62). This process exacerbates even further a sustained net of inflammatory actions that are associated with several inflammatory diseases such as arthritis, cancer, atherosclerosis, and inflammatory bowel disease (41, 62). Evidence also supports a link between AA, COX, and PGs and the classical “inflammatory” arm of the renin-angiotensin system (65–67). The renin-angiotensin system is an important physiological pathway involved in hypertension but is also associated with pleiotropic effects in the heart, brain, and muscle (65–67). Notably, hypertension—one of the most prevalent cardiovascular risk factors (21, 67)—is associated with inflammation, gut dysbiosis, physical impairment, and brain dysfunction (68).

Selectively targeting anti-inflammatory or inhibiting pro-inflammatory eicosanoid members may hold promise as a potential therapeutic strategy for managing cognitive and physical function (69, 70). For instance, in a well-characterized rodent model of aging, the Fisher344 x Brown Norway rat, the administration of a genetically modified probiotic with *Lactobacillus paracasei* secreting angiotensin (1–7) led to an acute and long-term overexpression of circulating levels of angiotensin (1–7, 71). The systemic overexpression of angiotensin (1–7)—a heptapeptide with vasodilatory characteristics—significantly decreased the expression of several pro-inflammatory markers including COX2, IL-1β, and TNF-α, with evidence of positive effects in brain function (71). Similarly, in an induced-obesity mouse model (i.e., the C57BL/6 strain), activation of lipoxin A4—a potent anti-inflammatory eicosanoid-derived member—led to decreased adipose inflammation while increasing Annexin-A1 (72). In a 5xFAD Alzheimer's disease mouse model, Annexin-A1—an anti-inflammatory glucocorticoid mediator in the peripheral system—promoted beneficial effects on amyloid-β clearance through the stimulation of amyloid-β phagocytosis by microglia (73). Collectively, these studies suggest the potential applicability of highly site-specific strategies to modulate eicosanoids, the microbiome, and the gut–brain axis, and hence potentially to target cognitive frailty.

## Phospholipids and Sphingolipids

Phospholipids are found primarily as glycerophospholipids and sphingolipids in the human diet (74). Sphingolipids differ from glycerophospholipids in that their chemical structure contains a long-chain aliphatic amino alcohol, the sphingoid base, while the phospholipids have the glycerol backbone (74). Both phospholipids and sphingolipids are characterized by great molecular diversity due to their linkage with other molecules such as ethanolamine, choline, inositol, and/or serine (41). As a result, these precursors lead to the production of phosphoinositides, phosphatidic acids, sphingosines, and ceramides (41).

Phospholipids and sphingolipids exert several pleiotropic effects on inflammation, vesicular trafficking, endocytosis, cell cycle and senescence, survival, and apoptosis, cell migration, and cell stress responses (75). Phospholipids and derivative molecules are more involved in pivotal aspects of cellular and tissue biology, including membrane shaping, cell growth, and apoptosis, and inflammatory cascades (16), being integral for gut barrier permeability (41). In turn, sphingolipids participate in numerous inflammatory processes but are more responsible for controlling intracellular trafficking and signaling, cell proliferation, adhesion, vascularization, and apoptosis mechanisms that are associated with immune-dependent and vascular-related chronic diseases (16, 41). For instance, ceramide—the basic structural unit of all sphingolipids—and ceramide-derivative enzymes are associated with the development and progression of inflammatory bowel disease (76). The activation of ceramides is implicated in response to metabolic endotoxemia due to the circulating elevation in LPS and several pro-inflammatory cytokines such as TNF-α and IL-1β (76). The overexpression on ceramide signaling also leads to adipose tissue inflammation and insulin resistance and is associated with obesity and type 2 diabetes (77). Brain disturbances of ceramide metabolism or sphingomyelinase—the enzyme that catalyzes the degradation of sphingomyelin to ceramide—are associated with the early stages of Alzheimer's and Parkinson's disease (78). Similarly, several other sphingolipid-derivative members are associated with obesity, type 2 diabetes, cancer, atherosclerosis, and rheumatoid arthritis (41, 79–82) but also gut dysbiosis (83).

Notably, Brown et al. (84) demonstrated in a mouse model that a lack of bacterial *Bacteroides-*derivative sphingolipids resulted in intestinal inflammation and altered the host ceramide pool (84). Furthermore, Brown et al. (84) also showed that sphingolipids derived from *Bacteroides*—a strain of the *Bacteroidetes* phylum (85)—are significantly decreased in the fecal samples of patients with inflammatory bowel disease, while host sphingolipids are increased and inversely associated with *Bacteroidetes* abundance (84). Collectively, these studies show that microbes can either directly (through their own sphingolipids) or indirectly (through the modulation of host sphingolipids) impact intestinal eubiosis, inflammation, gut permeability, and cognitive function. These studies suggest novel opportunities to utilize microbes or sphingolipid members as preventive or therapeutic strategies for managing inflammation, the gut–brain axis, and also cognitive function.

## Specialized Pro-resolving Lipid Mediators

The specialized pro-resolving lipid mediators (SPMs) are actively synthetized during acute inflammation from ω-6 AA or, even more, from the ω-3 PUFAs eicosapentaenoic acid (EPA), docosahexaenoic acid (DHA), and docosapentaenoic acid (DPA). The synthesis of these bioactive mediators occurs through the stereoselective and concerted action of the same enzymes engaged in classical eicosanoid production: COX, LOX, and cytochrome P450 (41). This group includes AA-derived lipoxins, EPA-derived resolvins (RvE1-3), the DHA-derived resolvins (RvD1-6), the protectin D1 (PD1), and maresins (MaR1 and MaR2) (43). Although many cell types can produce SPMs, the synthesis of these molecules occurs predominantly through the activation of immune cells such as neutrophils, monocytes, and macrophages (16, 86).

The SPMs stimulate the inflammation “resolution” signs, inducing cessation of leukocyte infiltration, recruitment, and stimulation of nonphlogistic mononuclear cells, promoting clearance of debris, infective pathogens and macrophages, and mediating efferocytosis (16, 86). In addition, these bioactive lipids inhibit proinflammatory cytokines by inducing the production of anti-inflammatory mediators, reducing the time of resolution, and promoting tissue regeneration, analgesia, and gain of function (16, 86).

What differentiates SPMs from other repair mediators is their role as immuno-resolvents and not as immune suppressors (87). The ability to enhance endogenous processes and recover homeostasis after an inflammatory response (88) indicates that SPMs are a promising therapeutic avenue. For instance, recent studies showed that SPMs may play an important role in the resolution of inflammation and intestinal mucosal epithelial repair (88). In a pre-clinical study in mouse surgery-induced cognitive decline, the activation of resolving D1 prevented neuronal dysfunction through the modulation of astrocyte activity and synaptic plasticity (89). This process protected rodent brains from neuroinflammation, synaptic dysfunction, and cognitive decline (89). In an *in vivo* model of LPS-induced inflammation, the activation of resolvin E1 attenuated mRNA levels of several pro-inflammatory cytokines and chemokines (IL-6 and monocyte chemoattractant protein-1) in C2C12 skeletal muscle myotubes, preventing skeletal muscle atrophy in this cell type (90). In an adult male Sprague-Dawley rat model, lipoxin A4—a known SPM with powerful anti-inflammatory functions—exerted beneficial effects in cognitive impairment induced by chronic cerebral hypoperfusion (91). The administration of lipoxin A4 methyl ester attenuated oxidative stress injury and reduced neuronal apoptosis (91). Overall, this promising evidence supports the applicability of these differentiated SPMs in many pathological mechanisms associated with the onset of cognitive and physical disfunction, although clinical evidence with human models is still lacking. Thus, evidence from well-designed randomized controlled trials is needed to confirm the efficacy of targeting SPMs as a therapeutic strategy for managing cognitive frailty.

## Endocannabinoids

The endocannabinoid family (eCB) is one complex system that assembles different ligands, analogs, and enzymes that are present in many organs and tissues including brain, liver, adipose tissue, muscle, pancreas, and gut microbiota (60, 61). Two of the most studied eCB ligands are the endogenous agonist *N*-arachidonoylethanolamide (AEA) and 2-arachidonoylglycerol (2-AG), which bind and activate type-1 and type-2 cannabinoid receptors (CB_1_ and CB_2_) (41, 60). The CB_1_ is mostly abundant in distinct areas of the brain as well as in peripheral nerve terminations, where it inhibits the release of neurotransmitters involved in sensory perception, memory processing, and motor activity (60). In contrast, CB_2_ is mainly expressed in peripheral organs, in lymphoid tissue and myeloid cells participating in immune response through the β and T lymphocytes (92).

The eCB system also includes their analogs: *O*-arachidonoylethanolamine, *N*-oleoylethanolamine (OEA), and *N*-palmitoylethanolamine (PEA) (60, 61, 93). These analogs are produced by most tissues and immune cells via a set of specific synthetizing enzymes, *N*-acylphosphatidylethanolamine-hydrolyzing phospholipase D (NAPE-PLD), which synthetizes AEA, and diacylglycerol lipase (DAGL), which synthetizes 2-AG. In turn, AEA is degraded by the fatty acid amide hydrolase (FAAH), while 2-AG is degraded by monoacylglycerol lipase (MAGL) (41, 93). These analogs enhance the effect of the endogenous eCB (i.e., AEA and 2-AG) by inhibiting their inactivation (60). These analogs can also activate other molecular targets including peroxisome proliferator-activated receptors (PPAR-α and PPAR-γ), the orphan G-protein coupled receptors (GPR55 and GPR119), and members of the transient receptor potential channels (e.g., vanilloid 1 receptors-TRPV1) (94). Physiologically, these molecular targets are present on nerve terminals of extrinsic primary afferents (TRPV1) and on the enteric nervous system and enterocytes (PPARα, GPR55) (93). They act “on demand” in the regulation of gastrointestinal motility, secretion, and the maintenance of the epithelial barrier integrity (60, 93, 95), and, critically, their imbalance is associated with several gastrointestinal diseases such as bowel inflammation and colon cancer (60, 93, 95).

The eCB system and its enzymes are among the most potent immunoregulatory compounds. They are able to regulate the functions of several cell sub-systems of either innate or adaptative immunity, such as monocytes/macrophages, dendritic cells, granulocytes, and T lymphocytes, with several members mostly exhibiting an anti-inflammatory action (41, 43). For instance, studies support the protective effect of PEA in several models of neurogenic inflammation, neuropathic pain, and neuroprotection in Parkinson's and Alzheimer's diseases (41). PEA is also considered a fat sensor, mediating the response to high-fat diets and regulating the thermogenic process through the activation of PPAR-α (96).

Altered endocannabinoid signaling is associated with changes in intestinal permeability, inflammation, and incretin release in human obesity (3, 17, 97). Changes in the gut microbiome composition also induce gut-barrier dysfunction leading to increase gut permeability (98). This process leads to leakage of gram-negative bacterial components, which elevates the plasmatic levels of LPS (i.e., metabolic endotoxemia), exacerbating the pro-inflammatory actions that are associated with the onset of several metabolic, inflammatory, and neurodegenerative disorders (60, 98). Due to the high abundance of the eCB receptors and enzymes in the central and peripheral nervous system, these mediators can rapidly react to disturbances in the gut to maintain homeostasis according to its physiological needs, functioning to reduce pain and to alleviate gastroenteric, neurodegenerative, and inflammatory damage (61, 92, 93). Thus, targeting the eCB system may be a promising therapeutic strategy to modulate the microbiome, the gut–brain axis, and potentially, cognitive frailty.

Collectively, these studies support the crosstalk between bioactive lipids, the brain, the gut, and the microbiome (16, 99). This complex network of signaling mediators that regulate cellular trafficking and signaling, cell structure, and energy storage is a promising therapeutic target for managing the gut–brain axis (16). Therapeutic interventions that selectively target anti-inflammatory bioactive lipids or, in turn, inhibit specific pro-inflammatory receptors or enzymes may hold promise for combating cognitive frailty.

## Targeting Bioactive Lipids as a Therapeutic Strategy for Cognitive Frailty

To date, there is no clinical evidence to support the applicability of bioactive lipids for affecting the gut–brain axis and its influence on cognitive frailty. Although, as previously outlined, evidence from multiple studies using bioactive lipids in chronic diseases with similar pathophysiological mechanisms highlight the applicability of these mediators for counteracting cognitive frailty. In this section, we will present potential therapeutic targets for managing cognitive frailty, focusing on the SPMs and the eCB system.

## Specialized Pro-resolving Lipid Mediators as Therapeutic Targets

Evidence from multiple studies supports the applicability of several members of SPMs for modulating cognitive and physical impairment. For instance, ω-3 DPA-derived protecting D1 is associated with significant improvements in weight recovery and rescue of cognitive deficit in a murine model of epilepsy (100). The intracerebroventricular injection of this signaling mediator dose-dependently reduced hippocampal expression of IL-1β and TNF-α mRNAs. Similarly, in a mouse model of surgery-induced cognitive decline, the activation of resolving D1 prevented neuronal dysfunction by modulating astrocyte activity and synaptic plasticity and protecting the brain from neuroinflammation, synaptic dysfunction, and cognitive decline (89). In an adult male Sprague-Dawley rat model, the administration of lipoxin A4 attenuated oxidative stress and reduced neuronal apoptosis, exerting beneficial effects in cognitive impairment (91). These studies support the use of SPM as a promising strategy for modulating inflammation and neuronal plasticity and reducing oxidative stress, which are important elements in neuronal protection.

In the context of inflammatory bowel disease, systemic treatment with ω-3 DPA-derived protectin D1 and ω-3 DPA-derived resolvin D5 protected against colitis and intestinal ischemia/reperfusion-induced inflammation in mice (101). In contrast, the inhibition of the activity of 15-lipoxygenase—one of the enzymes that mediates the synthesis of DPA-derived protectin D1—reduced the production of this anti-inflammatory SPM and, in turn, increased intestinal inflammation (101). These innovative strategies may be used to promote anti-inflammatory and tissue-protective effects in gut inflammatory disorders.

Several studies also support the use of SPMs as a modulation strategy to counterbalance skeletal muscle atrophy. For instance, in an *in vitro* study, the use of resolvin E1 attenuated the mRNA levels of several pro-inflammatory cytokines and chemokines in C2C12 skeletal muscle myotubes, preventing LPS-induced skeletal muscle atrophy (90). Moreover, supplementation in rodents with fish oil (rich in ω-3 PUFA) alleviated skeletal muscle atrophy during immobilization through attenuating disturbances in the activation of the Akt and p70-S6 protein kinases, muscle atrophy F-box, and muscle RING finger 1 gene expression, with partial preservation of myosin heavy chain content (102). Similarly, in cancer cachexia, the ingestion of EPA and DHA attenuated skeletal muscle protein catabolism through the inhibition of the ATP-ubiquitin-dependent proteolytic pathway, considered to play a major role in muscle catabolism in cachexia (103). Although the mechanisms by which EPA and DHA modulate their anti-inflammatory action on skeletal muscle are not completely clear, one potential mechanism is associated with the inhibition of the nuclear factor kappa β pathway, a transcriptional factor that up-regulates inflammatory gene expression (104). In contrast, the downregulation of this pathway decreases the expression of IL-1, TNF-α cytokines and the expression of genes encoding adhesion molecules responsible for leukocyte infiltration (104). This evidence suggests that boosting endogenous resolution responses in the brain, gut, and skeletal muscle may significantly improve inflammation, intestinal barrier integrity, and cognitive and physical impairment.

## Endocannabinoids as Therapeutic Targets

Several pharmacological, nutritional, and supplemental approaches have been explored for managing multiple diseases through the modulation of bioactive lipids receptors and enzymes. For instance, pharmacological approaches targeting CB_1_ resulted in a reduction in cardiometabolic risk and obesity (105). In multiple clinical trials (i.e., RIO Europe, RIO North America, and RIO Lipids), the downregulation of CB_1_ activity by SR141716 (i.e., rimonabant) reduced abdominal adiposity, fasting glucose levels, and cardiometabolic risk factors (105). Unfortunately, the downregulation of CB_1_ activity by a universally active CB_1_ inverse agonist elicited psychiatric side effects (e.g., anxiety, depression, and suicide) resulting in market withdrawal (60). Evidence from preclinical and clinical studies with a second generation of CB_1_ neutral antagonists and peripherally restricted CB_1_ inverse agonists (e.g., AM6545 and JD5037) resulted in similar metabolic benefits without the psychiatric side effects seen with rimonabant (106–110). Other pharmacological alternatives targeting other eCB family members used specific agonists of GPCR119 (e.g., APD-597, MBX-2982, GSK-1292263, and PSN821), an eCB receptor highly prevalent in enterocytes (111).

The modulation of specific eCB enzymes is also associated with positive effects in metabolic diseases. For instance, the NAPE-PLD enzyme is associated with fat absorption through the regulation of lipoprotein production in intestinal epithelial cells and with the control of OEA synthesis (112). The deletion of this enzyme in adipocytes is associated with obesity, inflammation, insulin resistance, glucose intolerance, and perturbed lipid metabolism (113). These changes are associated with a shift in gut microbiota composition and with alterations in the browning process in adipose tissue, decreasing the levels of the anti-inflammatory eCB receptors PEA and OEA (113). In contrast, peripheral administration of PEA in ovariectomized obese rats increases the expression of leptin receptors in the hypothalamus and is associated with anorectic and anti-inflammatory effects (96). These studies point to new approaches for cannabinoid-based therapy in obesity and metabolic disorders using specific GPCR agonists or NAPE-PLD enzyme inhibitors to mediate the production of AEA and 2-AG (60). Although obesity and metabolic disorders are associated with gut dysbiosis and degeneration of central nervous system (114), important pathological mechanisms in cognitive frailty (3), more research in human models is needed to confirm the applicability of eCB mediators.

In the context of neurodegenerative diseases, eCB agonists such as WIN-55 and 212-2 showed neuroprotective effects in Parkinson's disease due to their capacity to suppress excitotoxicity, glial activation, and oxidative injury (115). These eCB agonists also promoted additional benefits against bradykinesia and levodopa-induced dyskinesia—the motor characteristics of Parkinson's disease—by stimulating OEA synthesis (115). Remarkably, OEA and PEA have also shown protective effects against neuroinflammation, oxidative stress, and neurodegeneration in Alzheimer's disease (58, 116, 117). Physiologically, the amyloid precursor protein that forms amyloid-β plaques in Alzheimer's disease is commonly expressed in the enteric nervous system by gut bacteria (95). *Escherichia coli* and *Salmonella enterica* are among the many bacterial strains that express and secrete amyloid proteins and contribute to Alzheimer's disease (95). The activation of microglia induces the secretion of significant amounts of inflammatory nitric oxide synthase, elevating the production of nitrogen-reactive species that, in turn, increase oxidative injury and neurodegeneration (95). Notably, in a pre-clinical study with rats, the administration of a CB_2_ agonist, MDA7, provided neuronal protection, inhibiting amyloid-β fibril-induced microglial and astrocyte activation, promoting the clearance of amyloid-β, and attenuating synaptic plasticity deficits and learning and memory impairments (118). Despite these promising therapeutic results in pre-clinical studies in both diseases (54, 119), as extensively reviewed in Basavarajappa et al. (61), evidence in human models is still lacking.

The applicability of eCB agonists was also extended to cancer diseases, based on their known positive effects of reducing nausea and vomiting, both effects of chemotherapy, alleviating cancer pain, and stimulating appetite (120–122). Interestingly, one of the etiological mechanisms involved in the onset of cognitive frailty is anorexia of aging, as well as malnutrition (123). Evidence shows that lower dietary intake is linked to micronutrient deficiency, which, in turn, is associated with worse cognition and increased risk of frailty (123–125). In contrast, an adequate balance of daily calories protects neuronal integrity and cognitive function in the elderly (126). Thus, applying eCB agonists may be a promising therapeutic strategy for stimulating appetite in those that are at higher risk of anorexia of aging.

Remarkably, nutritional approaches such as western diets, characterized by a high ω-6:ω-3 PUFA ratio, can shift the eCB system and can realize the modulation of eCB tone by supplying high quantities of eCB precursors like AA, which, in turn, increases the synthesis of AA pro-inflammatory derivative members (127). In contrast, diet supplementation with DHA is associated with decreased levels of AA-derivative eCBs (e.g., AEA and 2-AG), reduced inflammation and fat mass, and improved glucose uptake in skeletal muscle in C57BL/6J mice (128). Collectively, these studies highlight the applicability of the eCB system as a promising therapeutic avenue for combating cognitive frailty, although evidence from clinical studies is still required to substantiate a causality and efficacy effect.

## Conclusion and Future Directions

**Figure 4:**
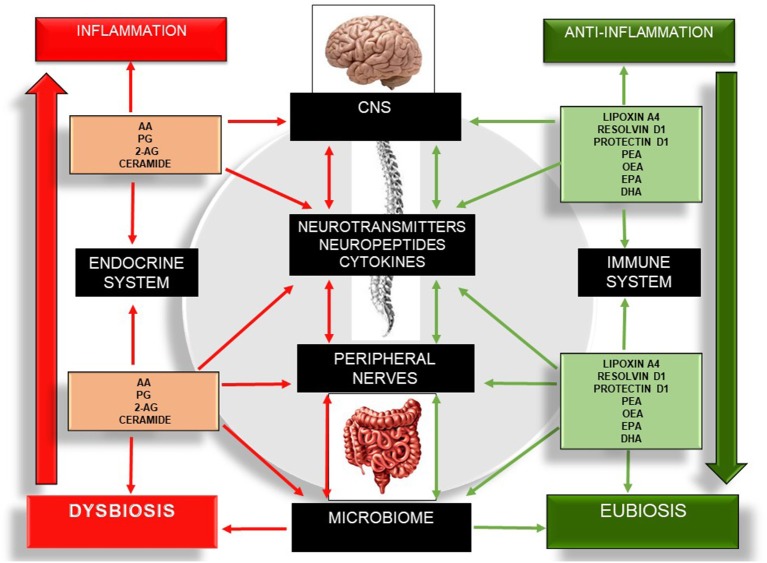
Schematic illustration of the bidirectional communication network involving the gut–brain axis and the different bioactive lipid members, as well as their roles in inflammation, the microbiome, and the expression of cytokines, neuropeptides, and neurotransmitters. Red arrows indicate pro-inflammation. Green arrows indicate anti-inflammation. The left side of the figure describes the pro-inflammatory actions of several bioactive lipids, which in turn drive a sustained net of inflammatory processes, increasing neuroinflammation, neurodegeneration, dysbiosis, and enteric and skeletal muscle tissue damage. The right side of the figure describes the anti-inflammatory effect of several bioactive lipids and their influences on the central nervous system, peripheral nerves, and microbiome. 2-AG, 2-Arachidonoylglycerol; CNS, Central Nervous System; DHA, Docosahexaenoic Acid; EPA, Eicosapentaenoid Acid; OEA, Oleoilethanolamide; PEA, Palmitoylethanolamine; PG, Prostaglandins.

Bioactive lipids regulate several metabolic and inflammatory processes and gut-barrier permeability and coordinate an integrative network of multi-cellular signaling mediators that are associated with physical and cognitive impairment (16). The ability of bioactive lipids to differentially modulate the brain, skeletal muscle, and gut microbiome is fascinating but is only now taking its “first steps.” Despite the number of interesting reviews (3, 16, 60, 64, 88, 92, 93, 98), to date, there have been no studies focusing on the interaction between these three components: gut microbiota, bioactive lipids, and cognitive frailty. Nonetheless, the current state of the art points to promising effects using different members of the SPM group and the eCB system on several chronic diseases. Therefore, the modulation of several SPM members and the eCB system may lead to potential routes for therapeutic development through the selective activation of anti-inflammatory bioactive lipids (lipoxins, resolvins, protectins, maresins, PEA, OEA) (64, 89–91, 117) or the inhibition/antagonism of their receptors and enzymes (Figure 4).

Despite the promising results outlined in this review, this evidence derives mostly from *in vitro* or animal studies associated with other age-related chronic diseases. The evidence supporting the applicability of SPMs and eCB in clinical studies is very limited. More research is needed to fully understand their effects and confirm their applicability in clinical practice, including physiological mechanisms, the acute and long-term effects, and particularly, side effects. There is evidence suggesting that the systematic activation of CB_1_ may lead to side effects such as cardiovascular dysfunction, digestion failure, neurological disorders, and potential for addiction (129). Thus, the potential applicability of the eCB system for managing cognitive frailty should be the first target of intense research to establish a causality and efficacy effect.

Finding more “holistic” interventions may also be a potential route to minimizing the side-effects seen with some pharmacological interventions. For instance, the traditional Mediterranean diet, characterized by the high rate of ω-3 PUFA derived from “fatty” fish (46), is associated with positive effects in cognitive and physical decline (130). Several systematic reviews and meta-analyses showed that adherence to a Mediterranean diet is associated with reduced risk of frailty, incident frailty, cognitive decline, mild cognitive impairment, Alzheimer's disease, and progression from mild cognitive impairment to Alzheimer's disease (131–135). A recent systematic review also showed that the Mediterranean diet is associated with myoprotective effects in several components of sarcopenia and declining physical function (136). Notably, sarcopenia is one of the main conditions involved in the onset of cognitive frailty. Thus, incorporating the Mediterranean diet in the daily habits of patients with cognitive frailty may be a useful strategy for increasing the amount of ω-3 PUFA precursors and, in turn, increasing the availability of anti-inflammatory ω-3 PUFA mediators (e.g., EPA and DHA) (137).

Although in this review we only focused on long-chain PUFA, there are many other bioactive lipid members with different structures associated with positive effects on cognitive and physical impairment. Among them are the short- and medium-chain fatty acids (SCFA and MCFA). SCFAs are mostly generated from fermentable fiber by colonic bacteria, whereas MCFAs are driven mostly from dietary triglycerides (e.g., milk and dairy products). High-fiber diets are associated with positive effects on the gut–brain axis in several chronic diseases (138, 139). Despite the positive metabolic and cognitive effects in several chronic conditions, the relationship between SCFAs and cognitive frailty is still understudied. To date, only one pre-clinical study associated high-fiber diets and SCFAs with age-associated muscle atrophy. In a pre-clinical study with aged female C57Bl/6 mice fed with a 5% sodium butyrate, Walsh et al. (140) showed that high-fiber diets have protective effects in neurogenic muscle atrophy through the reduction of histone deacetylase activity (140). When compared to a control diet, a sodium butyrate diet increased cross-sectional area of muscle fiber, prevented intramuscular fat accumulation, reduced fat mass, and improved glucose metabolism. The decrease in muscle atrophy was associated with increases in markers of mitochondrial biogenesis that led to improvements in oxidative stress, apoptosis, and antioxidant enzyme activity (140). This evidence indicates that a higher-fiber diet and, indirectly, increasing SCFA may be a promising intervention strategy for counteracting age-related physical impairment. Similarly, only a small number of pre-clinical studies confirm the neuroprotective effects of SCFA, MCFA, and high-fiber diets in cognitive frailty, as we and Kimura et al. (141) recently reviewed in depth elsewhere (138, 141).

Lastly, exercise has been found to induce biologically pro-resolution signs, involving the production of lipid mediators via cell–cell interactions with anti-inflammatory and pro-resolving bioactivity (142). As a result, these SPMs play important roles by limiting and potentially clearing myofiber damage and on the recruitment and polarization of monocytes to restore tissue homeostasis and facilitate muscle growth and regeneration (142). Thereby, targeting bioactive lipids, particularly the SPMs and the eCB system, may be a promising therapeutic approach to combat cognitive frailty. Nonetheless, more clinical studies are needed to confirm the crosstalk between the gut microbiome, gut–brain axis, and cognitive frailty and the potential applicability of these multiple pharmacological and non-pharmacological strategies as effective interventions for managing cognitive frailty.

## Author Contributions

LB wrote the draft of the manuscript. LB, YS, CC, and TB critically evaluated and revised the manuscript. All authors approved the final version of the manuscript for submission.

### Conflict of Interest

The authors declare that the research was conducted in the absence of any commercial or financial relationships that could be construed as a potential conflict of interest.

## Abbreviations

2-AG: 2-Arachidonoylglycerol
AA: Arachidonic Acid
AEA: Arachidonoylethanolamide
CB: Cannabinoid
CNS: Central Nervous System
COX: Cyclooxygenases
DAGL: Diacylglycerol Lipase
DHA: Docosahexaenoic Acid
eCB: Endocannabinoid
EPA: Eicosapentaenoid Acid
FAAH: Fatty Acid Amide Hydrolase
GPCR: G Protein Couple Receptor
HETE: Hydroxyeicosatetraenoids
IL: Interleukin
LOX: Lipoxygenases
LPS: Lipopolysaccharide
MAGL: Monoacylglycerol Lipase
NAAA: N-acylethanolamine Acid Amidase
NAE: N-acylethanolamine
NAPE-PLD: N-Acyl-Phosphatidylethanolamine-Hydrolyzing-Phospholipase D
OEA: Oleoilethanolamide
PEA: Palmitoylethanolamine
PG: Prostaglandins
PPAR: Peroxisome Proliferator-Activated Receptor
PUFA: Polyunsaturated Fatty Acids
RvD: DHA-derived Resolvins
RvE: EPA- derived Resolvin
SPM: Specialized Pro-resolving lipid Mediators
TLR: Toll-Like Receptor
TNF-α: Tumor Necrosis Factor- α.
